# Weaker Effects of Parental Education on Oral Nicotine Use of High School Students in Rural Areas: Marginalization-Related Diminished Returns

**DOI:** 10.31586/ojer.2025.6042

**Published:** 2025-03-20

**Authors:** Shervin Assari, Gandom Assari, Hossein Zare

**Affiliations:** 1 Department of Psychiatry, Charles R. Drew University of Medicine and Science, Los Angeles, CA, United States; 2 Department of Internal Medicine, Charles R. Drew University of Medicine and Science, Los Angeles, CA, United States; 3 Department of Family Medicine, Charles R. Drew University of Medicine and Science, Los Angeles, CA, United States; 4 Department of Urban Public Health, Charles R. Drew University of Medicine and Science, Los Angeles, CA, United States; 5 Marginalization-Related Diminished Returns (MDRs) Center, Los Angeles, CA, United States; 6 Canyon High School, Anaheim, CA, United States; 7 Department of Health Policy and Management, Johns Hopkins Bloomberg School of Public Health, Baltimore, MD, United States; 8 School of Business, University of Maryland Global Campus (UMGC), Adelphi, MD, United States

**Keywords:** Marginalization-Related Diminished Returns (MDRs), Nicotine Pouches, Adolescent Substance Use, Parental Education, Place-Based Marginalization, Monitoring the Future (MTF), Structural Equation Modeling (SEM)

## Abstract

**Background:**

Nicotine pouches, gummies, and candies have emerged as popular alternatives to traditional tobacco products among U.S. adolescents. While parental educational attainment is generally associated with youth substance use, marginalization-related diminished returns (MDRs) suggest that this effect may be weaker in marginalized populations, including non-Latino White communities. In particular, place-based marginalization—such as neighborhood economic disadvantage and school-level poverty—may attenuate the benefits of parental education. This study examines MDRs in the relationship between parental educational attainment and nicotine pouch/gummy/candy use among non-Latino White 12th graders in the 2024 Monitoring the Future (MTF) study.

**Methods:**

This study analyzed nationally representative data from the 2024 MTF study, focusing on non-Latino White 12th graders who reported parental education levels and adolescents’ use of nicotine pouch/gummy/candy. Structural equation modeling (SEM) was used to estimate the effects of parental education on adolescents’ use of nicotine pouches, gummies, and candies, while adjusting for demographic covariates. Place-based marginalization was operationalized using rural vs urban/suburban residence. Interaction terms tested whether the effect of parental education varied based on place of residence.

**Results:**

Higher parental educational attainment was associated with lower use of nicotine pouches, gummies, and candies. However, this effect was significantly weaker in rural areas.

**Conclusion:**

Public health interventions should account for place-based disparities rather than assuming a uniform effect of SES factors. This study highlights the need for policy responses that address structural inequities beyond individual family SES.

## 1. Introduction

The use of emerging nicotine products, such as nicotine pouches, gummies, and candies, has risen among adolescents in recent years, posing new challenges for public health and tobacco control efforts [1]. These products, often marketed as safer alternatives to traditional tobacco, are particularly appealing to youth due to their discreet nature, fruity flavors, and accessibility [1,2]. While the long-term health consequences of nicotine pouch/gummy/candy remain under investigation, early evidence suggests that they contribute to nicotine dependence, increase susceptibility to conventional tobacco use, and may have adverse neurodevelopmental effects in adolescents [3–5]. Given these concerns, understanding the social determinants of adolescents’ nicotine use is critical for informing prevention strategies and policy interventions [6].

Among various social determinants, parental education has historically been recognized as a key protective factor against substance use [7–9], with higher parental educational attainment generally associated with lower rates of smoking, vaping, and other risky behaviors among youth [7,10]. However, emerging research suggests that the protective benefits of parental education may not be equally distributed across all adolescents [11–19], including those experiencing place-based marginalization [20].

The concept of Marginalization-Related Diminished Returns (MDRs) [21] challenges traditional assumptions that higher parental socioeconomic status (SES) uniformly benefits all youth [22–24]. MDRs refer to the phenomenon where adolescents from marginalized backgrounds experience weaker protective effects of parental education on health-related behaviors compared to their more beneficial peers [25]. While MDRs have been extensively documented among racial and ethnic minority populations—demonstrating that Black, Latino, and American Indian adolescents receive fewer health and economic benefits from parental SES— [26] there is less literature on the role of geographic and place-based marginalization in shaping MDRs within non-Latino White populations. Neighborhood disadvantages, school poverty, and community-level stressors may erode the benefits of high parental education [20], thereby increasing the likelihood of risky health behaviors, including use of nicotine pouches, gummies, and candies.

Place-based marginalization manifests in various forms, including residential segregation, exposure to community stress, underfunded schools, and the targeted marketing of harmful substances in economically disadvantaged areas. Adolescents growing up in neighborhoods characterized by high poverty, social stressors, limited educational resources, and weakened social support networks may remain vulnerable, regardless of their family’s socioeconomic status. These environmental risk factors can erode the traditional advantages associated with having highly educated parents. Similarly, attending schools with concentrated poverty may limit academic engagement, increase exposure to peer substance use, and weaken the protective influence of parental monitoring. Collectively, these factors may reduce the buffering effects of parental education and contribute to increased susceptibility to nicotine products such as pouches, gummies, and candies among adolescents.

### Aims

While existing research on MDRs has primarily focused on racial and ethnic minority populations [26], this study extends the MDRs framework to non-Latino White adolescents, examining whether place-based disadvantage weakens the association between parental educational attainment and adolescents’ use of nicotine pouches, gummies, and candies. Using data from the 2024 Monitoring the Future (MTF) study [31–37], we investigate (1) whether parental education is inversely associated with adolescent use of nicotine pouches, gummies, and candies, (2) whether this protective association is weaker in marginalized environments, and (3) the potential mechanisms through which place-based factors mediate or moderate these effects. By employing Structural Equation Modeling (SEM) [38–45], we provide a nuanced analysis of these relationships, considering both direct and indirect pathways of influence.

This study contributes to the broader MDRs literature [21,46–55] by demonstrating that diminished returns of SES are not exclusive to racial and ethnic minority groups but also apply to non-Latino White adolescents living in marginalized environments. The findings have important implications for public health policies and interventions, underscoring the need to shift from individual-level approaches focused solely on parental education toward more structural interventions that address place-based inequalities. Understanding the complex interplay between SES, geography, and adolescent substance use can inform targeted policies aimed at reducing health disparities and promoting more equitable health outcomes.

## 2. Methods

### 2.1. Study Design and Sampling

This study is a secondary analysis of existing data with a cross-sectional design, utilizing the MTF-2024 dataset [31–37] on 12th graders.

### 2.2. Data Source

This study utilized data from the 2024 MTF study, a nationally representative survey assessing substance use behaviors and related attitudes among U.S. adolescents. The MTF study employs a multistage, stratified sampling design to ensure broad representation of high school students across the United States. The current analysis focused on 12th graders, examining factors associated with nicotine use.

### 2.3. Sample

The analytic sample consisted of 2,700 adolescents who completed the 2024 MTF survey and provided complete data on key variables, including adolescents’ use of nicotine pouch/gummy/candy, SES), and relevant covariates. Participants with missing data on these variables were excluded using listwise deletion or multiple imputation, depending on the level and pattern of missingness.

### 2.4. Measures

#### Use of Nicotine Pouches, Gummies, and Candies:

The primary outcome was past-month and past 12-month use of nicotine pouches, gummies, and candies. This was assessed through self-reported responses to survey items measuring various nicotine product use.

#### Socioeconomic Status (SES):

SES was operationalized using parental education as a proxy for economic resources.

#### Control Variables:

Additional covariates included age, gender, region of the country, and presence of two parents in the household, all of which have been linked to both parental education and adolescent substance use in prior literature.

### 2.5. Analytical Strategy

Structural Equation Modeling (SEM) [38,40,42,44,45,56,57] was employed to assess the direct and indirect effects of SES on adolescents’ use of nicotine pouch/gummy/candy, with a specific focus on MDRs. The SEM framework allowed for simultaneous estimation of multiple relationships while accounting for measurement error. Model fit was evaluated using standard model fit indices, including Comparative Fit Index (CFI), Tucker-Lewis Index (TLI), and Root Mean Square Error of Approximation (RMSEA) [57–59]. To test for differences in the association between parental SES and adolescents’ use of nicotine pouches, gummies, and candies based on rural versus urban/suburban residence, multi-group SEM was conducted, comparing paths across these groups. Wald tests and moderation analyses were used to determine whether the effects of SES on adolescents’ nicotine use varied by rurality. Missing data were addressed using Full Information Maximum Likelihood (FIML) [60, 61] estimation to minimize bias. All analyses were conducted using Stata 18.0, with complex survey weights applied to adjust for the MTF study’s sampling design. Statistical significance was set at p < 0.05.

## 3. Results

**Figure 1.**
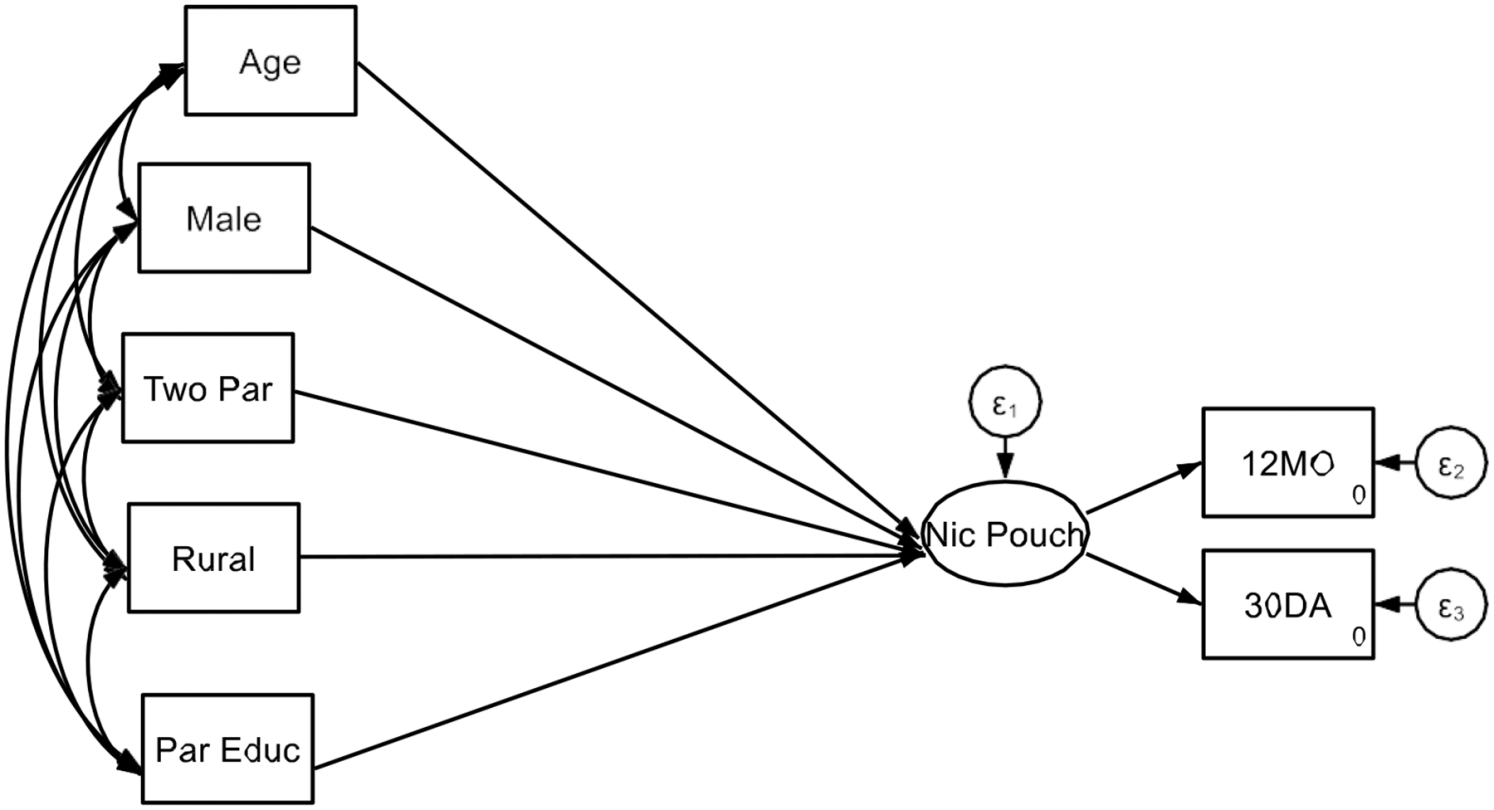
Structural Equation Model 1 Without Interaction

Table 1 (Figure 1) presents the results of Structural Equation Model 1, which examines the association between various sociodemographic factors and adolescents’ frequency of nicotine pouch, gummy, and candy use. Age was significantly associated with higher nicotine use, with adolescents over 18 years old reporting greater frequency of use (B = 0.20, SE = 0.02, p < 0.001). Sex also played a role, as males had a significantly higher likelihood of using nicotine pouches, gummies, and candies compared to females (B = 0.24, SE = 0.03, p < 0.001). Family structure was another significant factor, with adolescents living in two-parent households exhibiting higher frequency of nicotine use than those in single-parent or other household structures (B = 0.16, SE = 0.03, p < 0.001). Similarly, rural residence was associated with increased use of nicotine products (B = 0.15, SE = 0.03, p < 0.001), suggesting that adolescents in rural areas may have greater exposure to or accessibility of these products. Socioeconomic status (SES), measured by the highest level of parental education, was positively associated with nicotine use (B = 0.24, SE = 0.02, p < 0.001), indicating that adolescents from higher SES backgrounds reported greater frequency of nicotine pouch, gummy, and candy use.

Table 2 presents the results of Structural Equation Model 2 with interaction, examining how SES, rural residence, and their interaction influence adolescents’ frequency of nicotine pouch, gummy, and candy use. Consistent with prior models, age, sex, and family structure were significant predictors of nicotine use. Older adolescents (>18 years) exhibited a higher frequency of use (B = 0.18, SE = 0.02, p < 0.001), and males were more likely to use these nicotine products than females (B = 0.22, SE = 0.02, p < 0.001). Additionally, adolescents living in two-parent households had significantly higher nicotine use compared to those in single-parent or other household structures (B = 0.22, SE = 0.02, p < 0.001).

A key finding was the significant positive association between rural residence and nicotine use. Adolescents residing in rural areas reported significantly higher frequency of nicotine pouch, gummy, and candy use compared to their urban/suburban counterparts (B = 0.38, SE = 0.10, p < 0.001). This suggests that rural environments may contribute to greater exposure to or availability of these products.

Interestingly, SES (parental education) alone was negatively associated with nicotine use (B = −0.30, SE = 0.10, p = 0.002), indicating that adolescents with more highly educated parents reported lower nicotine use overall. However, this effect varied based on rural residence, as demonstrated by the significant SES × rural area interaction (B = 0.23, SE = 0.02, p < 0.001). This interaction suggests that the effect of parental education on nicotine use was weaker in rural areas.

Table 3 presents the results of the multi-group (stratified by rurality) Structural Equation Model, which examines the associations between sociodemographic factors and adolescents’ frequency of nicotine pouch, gummy, and candy-use separately for urban/suburban and rural adolescents.

### 3.1. Urban/Suburban Adolescents

Among adolescents residing in urban and suburban areas, several factors were significantly associated with nicotine use. Older age (>18 years) was linked to increased nicotine use (B = 0.19, SE = 0.03, p < 0.001), as was male sex (B = 0.23, SE = 0.03, p < 0.001). Additionally, living in a two-parent household was positively associated with nicotine use (B = 0.18, SE = 0.03, p < 0.001). Notably, higher parental education (SES) was positively associated with nicotine use in urban/suburban adolescents (B = 0.28, SE = 0.03, p < 0.001), suggesting that in these areas, adolescents from higher SES backgrounds had greater nicotine pouch, gummy, and candy use. This finding may indicate increased exposure to emerging nicotine products within higher-income communities, potentially due to differential marketing, peer influences, or access to disposable income.

### 3.2. Rural Adolescents

For rural adolescents, the patterns were somewhat different. As in urban/suburban areas, older age (B = 0.22, SE = 0.05, p < 0.001) and male sex (B = 0.24, SE = 0.06, p < 0.001) were associated with higher nicotine use. However, the effect of living in a two-parent household was weaker in rural areas (B = 0.12, SE = 0.06, p < 0.001), indicating that family structure may play a diminished role in these environments. The association between SES (parental education) and nicotine use was also weaker in rural adolescents (B = 0.18, SE = 0.04, p < 0.001) compared to their urban/suburban peers (B = 0.28, SE = 0.03, p < 0.001). This finding aligns with the MDRs framework, which suggests that the effects of higher SES are attenuated in certain contexts, such as rural settings.

## 4. Discussion

The findings of this study support the MDRs framework, demonstrating that the effect of parental education on adolescents’ use of nicotine pouch/gummy/candy is weaker for non-Latino White youth in marginalized environments. While higher parental education was generally associated with a lower likelihood of nicotine use, this association was significantly attenuated among adolescents from disadvantaged neighborhoods and schools. These results highlight the need to reconsider conventional SES-based assumptions in public health, emphasizing the role of environmental and structural factors in shaping adolescent health behaviors.

The primary goal of this study was to examine whether parental education predicts non-Latino White adolescents from using nicotine pouch/gummy/candy, and whether this effect is modified by place-based marginalization. Using data from the 2024 MTF study, we found that while adolescents with highly educated parents were less likely to use nicotine pouch/gummy/candy overall, this effect was significantly weaker for those living in high-poverty neighborhoods and attending under-resourced schools. Our SEM revealed that place-based variation in the relationship between parental education and adolescents’ use of nicotine pouch/gummy/candy, suggesting that adolescents in marginalized environments face additional barriers that reduce the effectiveness of parental SES advantages.

The MDRs framework posits that social and economic resources tend to yield fewer outcomes for individuals who are socially marginalized [51,53–55,62–66]. While prior research has largely focused on MDRs among racial and ethnic minority groups [51,53–55,62–66], our findings demonstrate that MDRs also apply within non-Latino White populations exposed to structural disadvantage. Even among White adolescents, those in high-poverty areas benefit less from parental education due to environmental stressors, exposure to tobacco marketing, and reduced access to supportive community resources.

Place-based factors play a crucial role in shaping MDRs, as neighborhood and school-level SES directly influence adolescent behavior. The weaker effect of parental education in marginalized areas suggests that geographic location can amplify disparities in health and substance use outcomes. Adolescents in low resource neighborhoods may face greater exposure to stress, fewer extracurricular opportunities, and weaker institutional support systems, all of which contribute to increased substance use risk. Furthermore, the tobacco industry has been known to target economically disadvantaged communities with aggressive marketing strategies, making nicotine products more accessible in high-risk areas [67–69]. These geographic disparities suggest that public health efforts must adopt a place-based approach to address structural inequities in tobacco control.

The weaker protective effect of parental education on tobacco use among 12th graders in rural areas compared to urban and suburban areas may be attributed to several structural and contextual factors. Rural environments often have higher tobacco acceptability, greater access to tobacco products, and fewer regulatory restrictions, which may weaken the role of parental education in discouraging tobacco use. Additionally, rural youth may experience different social influences, including stronger peer and community norms favoring tobacco use, which can diminish the impact of parental educational attainment. Economic stressors and limited educational and occupational opportunities in rural settings may also reduce the perceived benefits of higher parental education, making youth less responsive to the protective effects associated with their parents’ educational background. Furthermore, rural areas often have fewer health education resources and anti-tobacco campaigns, which in urban and suburban areas may reinforce the influence of parental education on adolescent tobacco behaviors. These combined factors contribute to the diminished protective role of parental education in preventing tobacco use among rural youth.

### 4.1. Policy Implications

The results of this study have important policy implications. Traditional tobacco prevention programs often focus on individual-level factors such as parental education, assuming that higher SES has a universal effect on substance use. However, our findings indicate that structural and environmental factors must also be addressed to reduce adolescents’ use of nicotine pouch/gummy/candy. Tobacco control policies should prioritize geographic equity by implementing stronger advertising restrictions, increasing school-based prevention programs, and addressing community-level economic disparities. Additionally, public health campaigns should incorporate targeted interventions for high-poverty schools and neighborhoods rather than relying solely on family-based educational strategies.

Disparities in social and health outcomes are shaped by at least two interrelated mechanisms: differential access to socioeconomic resources [70–76] and MDRs [11–19]. The first mechanism, lower socioeconomic resources, highlights how historically marginalized groups often have reduced access to wealth, high-quality education, stable employment, and healthcare, leading to persistent inequalities. Structural barriers such as residential segregation, underfunded schools, and employment discrimination limit opportunities for upward mobility, resulting in lower average SES among minoritized populations. However, disparities are not fully explained by differences in SES alone, as the second mechanism, diminished returns, further exacerbates inequalities. Even when individuals from minoritized backgrounds attain high levels of education or income, they tend to receive fewer benefits from these resources compared to their White counterparts. MDRs manifest in multiple domains, such as weaker associations between education and income, wealth accumulation, and health outcomes among racially and ethnically marginalized populations.

A recent study [77] explored the relationship between parental education and aspirations for graduate or professional education among non-Latino White adolescents, with a particular emphasis on urban-suburban versus rural differences. Utilizing data from the 12th-grade cohort of the MTF 2024 survey, the study was grounded in the Motivational Theory of Life-Span Development, which posits that educational aspirations are shaped by both internal and external resources. While parental education is a well-established predictor of academic aspirations, its influence may vary by geographic context, reflecting spatial patterns of MDRs. To investigate these dynamics, the study conducted multivariate analyses assessing the association between parental education and aspirations for graduate or professional education. The analysis further examined whether this relationship was moderated by geographic location (urban-suburban vs. rural) to identify place-based MDRs. Results indicated that higher parental education was associated with greater aspirations for advanced education, yet this effect was weaker in rural areas compared to urban and suburban settings. These findings suggested that even among non-Latino White adolescents, rural residence attenuates the benefits of socioeconomic resources, providing empirical support for place-based MDRs. The authors concluded that rural youth experience a dual disadvantage—facing both lower overall socioeconomic status and weaker returns on educational resources. To address disparities in educational aspirations in rural areas, policymakers should go beyond resource allocation and focus on enhancing the effectiveness of educational opportunities, ensuring that socioeconomic advantages translate into equitable academic outcomes across geographic settings.

Addressing disparities, therefore, requires not only increasing access to high-quality education and economic resources but also ensuring that these resources translate into meaningful gains. For example, policies that expand job opportunities and promote workplace equity are crucial for reducing MDRs by ensuring that higher education leads to comparable economic stability across racial and ethnic groups. Without addressing both mechanisms—resource access and diminished returns—efforts to reduce disparities will remain insufficient.

Ignoring MDRs in policy design and implementation risks exacerbating disparities, as universal solutions may unintentionally widen the gap. When resources and opportunities are made universally available, they often generate greater benefits for groups with preexisting structural advantages—such as urban residents—who can more effectively capitalize on these opportunities. In contrast, marginalized groups may face systemic barriers that limit their ability to fully benefit from the same resources, leading to an uneven distribution of advantages. This phenomenon can result in iatrogenic effects, where well-intended policies designed to reduce disparities inadvertently reinforce or deepen existing inequalities.

For instance, if a policy aims to improve educational access by increasing funding for all public schools, wealthier urban districts with greater institutional capacity may benefit more than under-resourced rural or predominantly minoritized schools, thereby worsening inequities. Similarly, job creation initiatives that do not account for hiring discrimination or occupational segregation may disproportionately benefit already advantaged populations while leaving minoritized groups behind.

To mitigate these unintended consequences, policies must be accompanied by rigorous evaluations that explicitly assess differential returns across social groups. This requires monitoring whether geographically marginalized populations are deriving benefits at rates comparable to more geographically resourced groups and making necessary adjustments when disparities in return emerge. Targeted interventions—such as equity-focused hiring practices, affirmative resource distribution, and structural reforms—should complement universal policies to ensure that benefits reach those who need them most. Without such intentional oversight, policies that overlook MDRs risk perpetuating systemic inequalities rather than dismantling them.

These findings should not be interpreted as a justification for reducing investments in rural areas simply because their return on investment appears smaller. Rather, they highlight the need for sustained and strategic investment to ensure that resources yield comparable outcomes for rural communities as they do for urban populations. Lower returns on investment in rural areas often reflect systemic barriers—such as limited infrastructure, fewer employment opportunities, weaker healthcare access, and lower-quality education—rather than inefficiencies in investment itself. Instead of scaling back efforts, policymakers should increase and sustain comprehensive investments that address these structural limitations. This includes expanding transportation networks, increasing broadband internet access, improving educational quality, and incentivizing businesses to create jobs in rural regions. By doing so, policymakers can foster equity-driven solutions, ensuring that rural residents have the same opportunities to benefit from economic and social resources as their urban counterparts. The goal should not be to reallocate resources away from rural areas but to reduce disparities in the effectiveness of these investments, ensuring that all communities—regardless of geography—achieve meaningful and sustainable progress.

It is essential to emphasize that the observed weaker returns on investment in rural areas are shaped by structural and social forces that systematically limit opportunities for rural populations. Factors such as geographic isolation, residential segregation, limited access to high-quality education, weaker job markets, inadequate public transportation, and reduced healthcare infrastructure all contribute to the diminished effectiveness of resources in these areas.

For example, even when rural residents attain higher levels of education, their job prospects may remain constrained due to the absence of industries that match their skill sets. Similarly, limited transportation options may prevent individuals from accessing higher-paying jobs in urban labor markets, further weakening the returns on education and training. These constraints do not reflect individual capability but rather systemic barriers that hinder the full realization of human potential in rural areas.

Therefore, policy solutions must address these structural limitations by investing in infrastructure, expanding economic opportunities, and improving access to critical services [78]. By doing so, rural communities can equitably benefit from social and economic resources, reducing disparities and fostering long-term well-being for all populations, regardless of geographic location.

### 4.2. Limitations

Several limitations of our study should be noted. The cross-sectional nature of the MTF survey data limits the ability to make causal inferences, as it only captures associations at a single point in time. Additionally, self-reported use of nicotine pouch/gummy/candy may be subject to reporting bias, potentially leading to underestimation or overestimation of substance use behaviors. Future studies should incorporate objective measures of nicotine use and other substance use indicators to enhance the reliability and validity of findings. Studies may also use multi-informants and include a wider range of nicotine products. Our study also did not include other risk factors of substance use such as substance availability, parental use, mental health problems, or environmental risks such as school environments, and peer and parental influences.

### 4.3. Strengths

One of the key strengths of this study is its use of nationally representative data from the 2024 MTF study, which enhances the generalizability of findings to U.S. adolescents. Additionally, the application of SEM allowed for a sophisticated analysis of both direct and indirect effects, providing a more nuanced understanding of MDRs in adolescent substance use.

### 4.4. Future Research Directions

Future research should explore the relationships between SES, rurality, and adolescent substance use longitudinally. Changes in adolescents’ use of nicotine pouch/gummy/candy and changes in family SES should be mapped over a period of time. Investigating other social determinants, such as neighborhood conditions and wealth could further elucidate the mechanisms contributing to MDRs. Additionally, studies should examine how policy changes, such as tobacco regulations and marketing restrictions, differentially impact adolescents’ substance use from various racial, socioeconomic, and geographic backgrounds. Incorporating mixed methods studies with qualitative components could also provide deeper insights into the lived experiences of rural, urban/suburban adolescents navigating these disparities.

## 5. Conclusion

This study highlights that place-based marginalization substantially weakens the effect of parental education on adolescents’ use of nicotine pouches, gummies, and candies, underscoring the role of structural determinants in shaping health disparities. The findings suggest that MDRs extend beyond racial and ethnic minority groups, also affecting populations in resource-limited environments. Addressing these geographic inequities in health outcomes requires a shift from individual-focused interventions to systemic policies that mitigate place-based disparities in substance use risk.

## Figures and Tables

**Figure 2.**
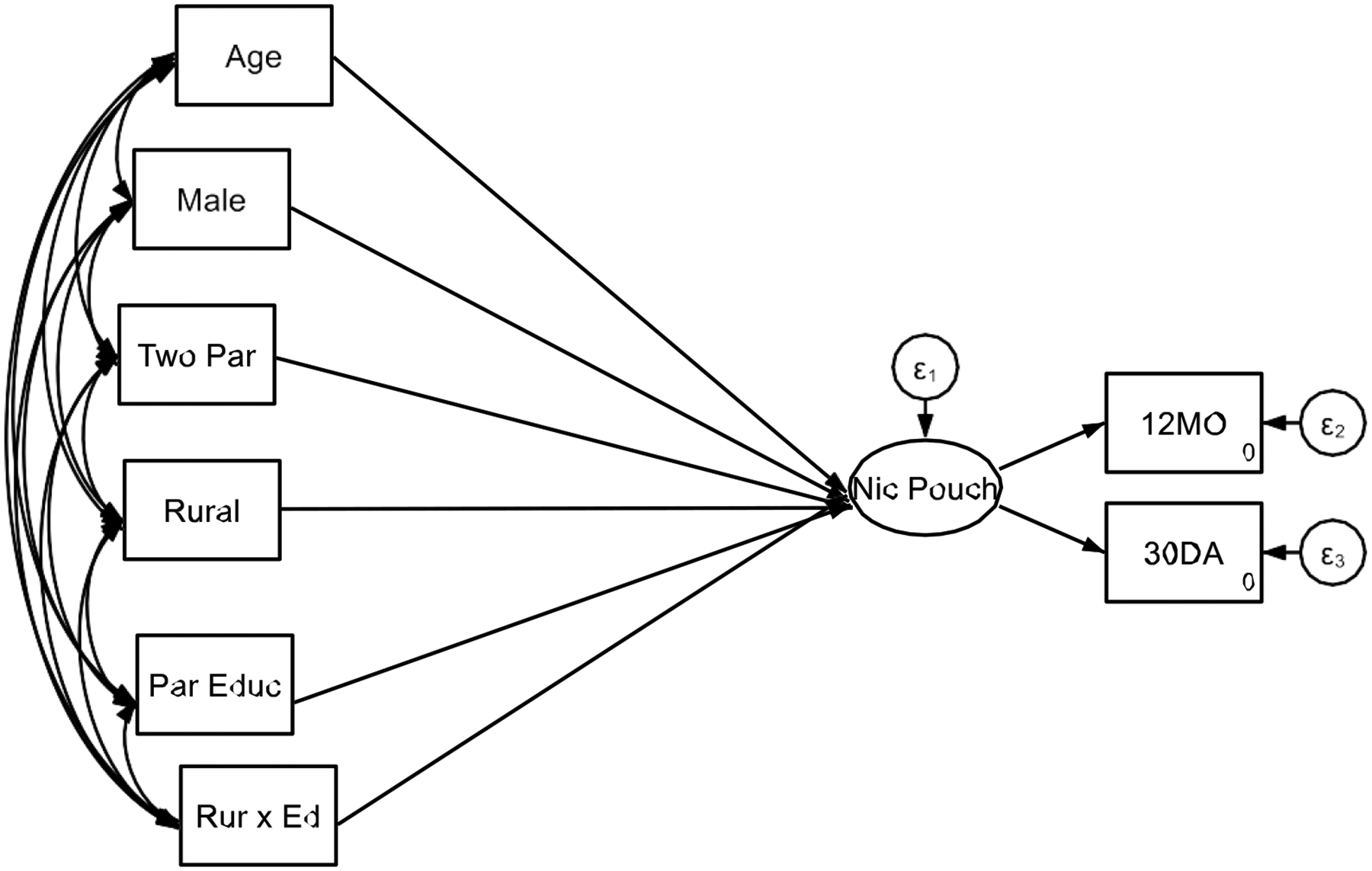
Results of Structural Equation Model 2 With Interaction

**Figure 3.**
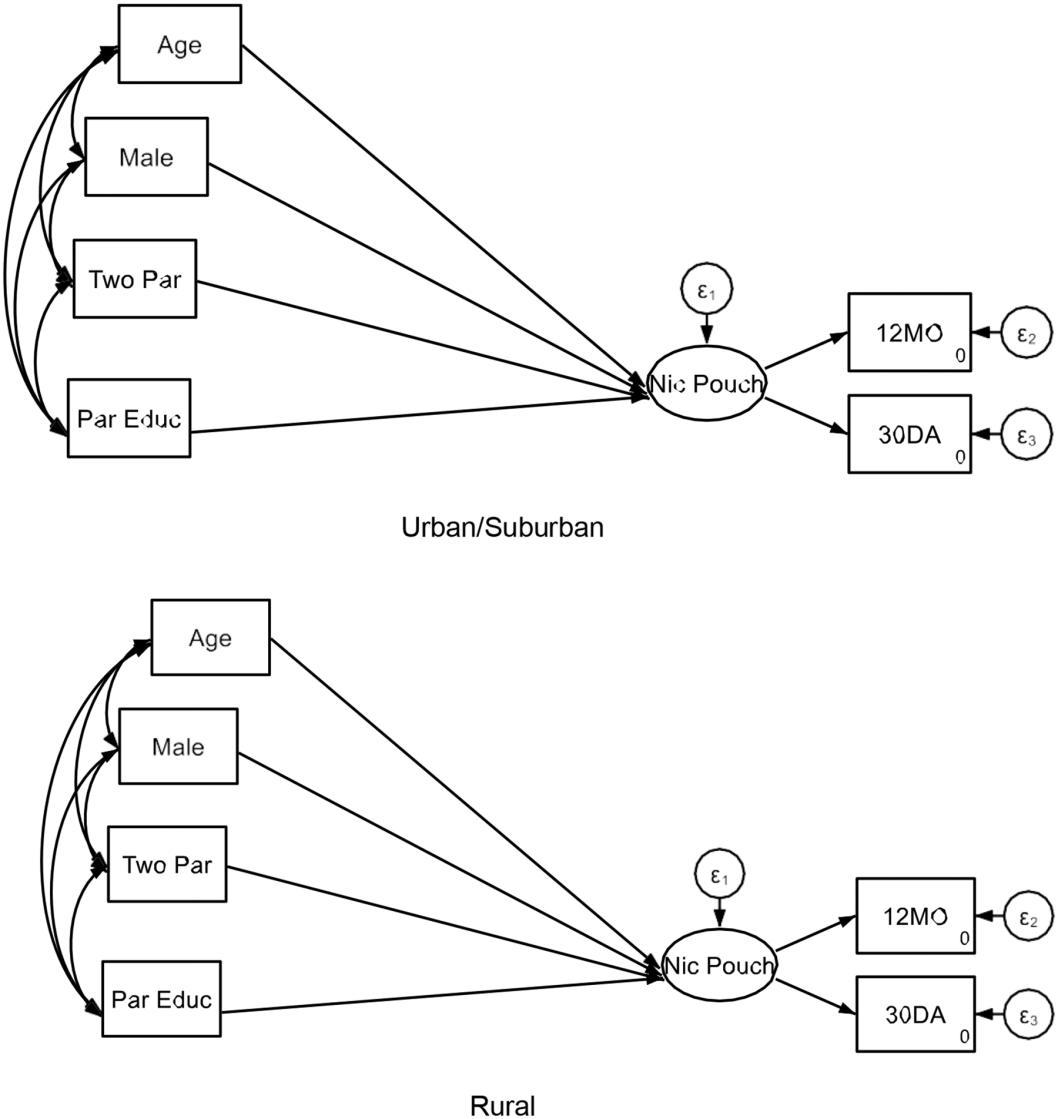
Multi-Group Structural Equation Model 3 Based on Rural Residence

**Table 1.** Results of Structural Equation Model 1 Without Interaction

	B	SE	95%	CI	p
Age (>18 Years)	0.20	0.02	0.15	0.24	< 0.001
Sex (Male)	0.24	0.03	0.18	0.29	< 0.001
Two Parents in the Household	0.16	0.03	0.11	0.22	< 0.001
Rural Area	0.15	0.03	0.10	0.20	< 0.001
SES (Max Parental Education; 1–6)	0.24	0.02	0.20	0.28	< 0.001

Note: Outcome: Adolescents’ Frequency of Use of Nicotine Pouch/Gummy/Candy

**Table 2.** Results of Structural Equation Model 2 With Interaction

	B	SE	95%	CI	p
Age (>18)	0.18	0.02	0.14	0.22	< 0.001
Sex (Male)	0.22	0.02	0.18	0.26	< 0.001
Two Parents in the Household	0.22	0.02	0.17	0.26	< 0.001
Rural Area	0.38	0.10	0.19	0.57	< 0.001
SES (Max Parental Education; 1–6)	−0.30	0.10	−0.49	−0.11	0.002
SES (Max Parental Education (1–6) x Rural Area	0.23	0.02	0.19	0.27	< 0.001

Note: Outcome: Adolescents’ Frequency of Use of Nicotine Pouch/Gummy/Candy

**Table 3.** Results of Multi-Group (Stratified by Rurality) Structural Equation Model

	B	SE	95%	CI	p
**Urban/Suburban**					
Age (>18)	0.19	0.03	0.14	0.25	< 0.001
Sex (Male)	0.23	0.03	0.17	0.29	< 0.001
Two Parents in the Household	0.18	0.03	0.11	0.24	< 0.001
SES (Max Parental Education; 1–6)	0.28	0.03	0.23	0.33	< 0.001
**Rural**					
Age (>18)	0.22	0.05	0.12	0.31	< 0.001
Sex (Male)	0.24	0.06	0.13	0.36	< 0.001
Two Parents in the Household	0.12	0.06	0.00	0.24	< 0.001
SES (Max Parental Education; 1–6)	0.18	0.04	0.09	0.27	< 0.001

Note: Outcome: Adolescents’ Frequency of Use of Nicotine Pouch/Gummy/Candy

## Data Availability

The MTF data used in this study were publicly available and downloaded from the Inter-university Consortium for Political and Social Research (ICPSR) at the University of Michigan.
